# Detecting modular brain states in rest and task

**DOI:** 10.1162/netn_a_00090

**Published:** 2019-07-01

**Authors:** Aya Kabbara, Mohamad Khalil, Georges O’Neill, Kathy Dujardin, Youssof El Traboulsi, Fabrice Wendling, Mahmoud Hassan

**Affiliations:** Azm Center for Research in Biotechnology and Its Applications, EDST, Lebanese University, Beirut, Lebanon; University of Rennes, LTSI - U1099, Rennes, France; CRSI Lab, Engineering Faculty, Lebanese University, Beirut, Lebanon; Azm Center for Research in Biotechnology and Its Applications, EDST, Lebanese University, Beirut, Lebanon; CRSI Lab, Engineering Faculty, Lebanese University, Beirut, Lebanon; Sir Peter Mansfield Imaging Centre, School of Physics and Astronomy, University of Nottingham, University Park, Nottingham, United Kingdom; INSERM, U1171, Lille, France; CHU Lille, Neurology and Movement Disorders Department, Lille, France; LaMA-Liban, Lebanese University, Tripoli, Lebanon; University of Rennes, LTSI - U1099, Rennes, France; University of Rennes, LTSI - U1099, Rennes, France

**Keywords:** Brain network dynamics, Functional connectivity, M/EEG source-space networks

## Abstract

The human brain is a dynamic networked system that continually reconfigures its functional connectivity patterns over time. Thus, developing approaches able to adequately detect fast brain dynamics is critical. Of particular interest are the methods that analyze the modular structure of brain networks, that is, the presence of clusters of regions that are densely interconnected. In this paper, we propose a novel framework to identify fast modular states that dynamically fluctuate over time during rest and task. We started by demonstrating the feasibility and relevance of this framework using simulated data. Compared with other methods, our algorithm was able to identify the simulated networks with high temporal and spatial accuracies. We further applied the proposed framework on MEG data recorded during a finger movement task, identifying modular states linking somatosensory and primary motor regions. The algorithm was also performed on dense-EEG data recorded during a picture naming task, revealing the subsecond transition between several modular states that relate to visual processing, semantic processing, and language. Next, we tested our method on a dataset of resting-state dense-EEG signals recorded from 124 patients with Parkinson’s disease. Results disclosed brain modular states that differentiate cognitively intact patients, patients with moderate cognitive deficits, and patients with severe cognitive deficits. Our new approach complements classical methods, offering a new way to track the brain modular states, in healthy subjects and patients, on an adequate task-specific timescale.

## Author Summary

The brain is a dynamic modular network. Thus, exploring the dynamic behavior of the brain can reveal insights about its characteristics during rest and task, in healthy and pathological conditions. In this paper, we propose a new framework that aims to track the dynamic changes of the modular brain organization. The method presents two algorithms that can be applied during task-free or task-related paradigms. Using simulations, we demonstrated the advantages of the proposed algorithm over existing methods. The proposed algorithm was also tested on three datasets recorded from real EEG and MEG acquisitions. Overall, results showed the capacity of the proposed algorithm to track brain network dynamics with good spatial and temporal accuracy.

## INTRODUCTION

The human brain is a modular dynamic system. Following fast neuronal activity (Pfurtscheller & Aranibar, 1977; Pfurtscheller & Lopes Da Silva, 1999), the spatiotemporal organization of resting (Baker et al., 2014; Damaraju et al., 2014; de Pasquale et al., 2012; de Pasquale, Della Penna, Sporns, Romani, & Corbetta, 2015; Kabbara, El Falou, Khalil, Wendling, & Hassan, 2017) and task-evoked connectivity (Bola & Sabel, 2015; Hassan, Benquet, et al., 2015; Hutchison et al., 2013; O’Neill, Tewarie, Colclough, et al., 2017) is in constant flux.

Hence, an appropriate description of time-varying connectivity is of utmost importance to understand how cognitive and behavioral functions are supported by networks. In this context, several frameworks have been suggested to explore the time-varying nature of functional brain connectivity. Among them, hidden Markov model approaches (Baker et al., 2014; Vidaurre et al., 2017), K-means clustering (Allen, Damaraju, Eichele, Wu, & Calhoun, 2017; Allen et al., 2014; Damaraju et al., 2014), independent component analysis (ICA; O’Neill, Tewarie, Colclough, et al., 2017), principal component analysis (Preti & Van De Ville, 2016), or orthogonal connectivity factorization (Hyvärinen, Hirayama, Kiviniemi, & Kawanabe, 2016) have been applied to identify the main brain networks shaping dynamic functional connectivity. These methods consist of grouping the temporal networks into states, where each state reflects a unique spatial connectivity pattern. It is important to note that in these frameworks, states are identified without looking at the modular organization of the networks.

However, because of the modular organization of the human brain network (Sporns & Betzel, 2016) methods for detecting network communities (or modules) are of particular interest (Sporns & Betzel, 2016). These methods decompose the network into building blocks or modules that are internally strongly connected, often corresponding to specialized functions. Importantly, during a learning task, Basset et al. showed that the flexibility (defined as how often a given node changes its modular affiliation over time) of the networks facilitates the prediction of individual future performances in next learning sessions (Bassett et al., 2011), remembering, attention, and integrated reasoning (Gallen et al., 2016).

To compute modularity in networks collected across multiple slices (time points), one could simply decompose each network independently. Another algorithm proposed to link corresponding nodes across slices before generating communities. This algorithm is known as multislice modularity (also called multilayer modularity; Mucha, Richardson, Macon, Porter, & Onnela, 2010), which has been used to follow changes of the modular architecture in different applications (Bassett et al., 2011; Bassett, Yang, Wymbs, & Grafton, 2015). However, the problem with solely applying these techniques is that they may not lead to easily interpretable results because of the particularly high number of modular structures generated. More specifically, the dynamic modular structures obtained are technically difficult to visualize since their number is equal to the number of slices. Alternatively, one could use the multislice modularity algorithm to generate a community structure consistent over time. But such a strategy will constrain the dynamic analysis. Hence, it would be very useful to find the main modular structures driving the dynamics of neural activity. To date, there is no automatic framework that is able to decipher modular brain states, that is, subsets of brain modules implicated in a given brain function at a given time period.

**Figure 1.**
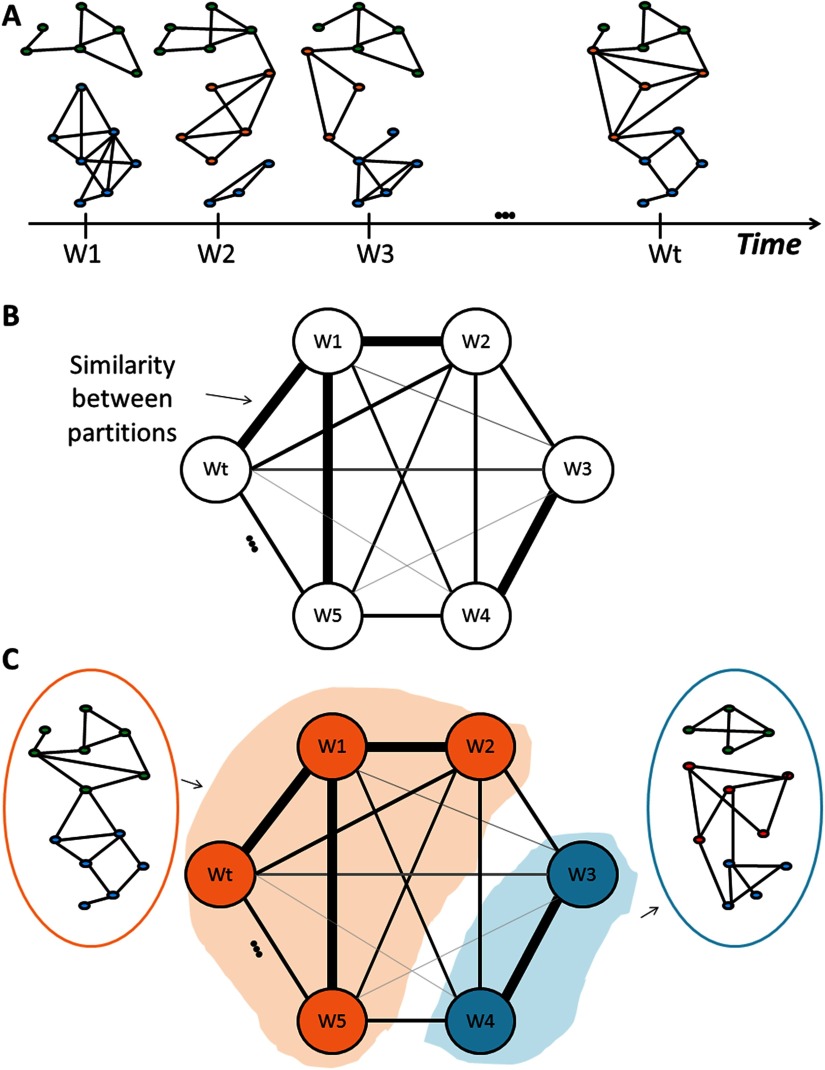
The algorithm procedure. (A) Computation of modules for each temporal network (Wt). (B) Assessment of the similarity between the dynamic modular structures. (C) Clustering the similarity matrix into “categorical modules.”

Here, we propose a novel framework aiming to elucidate the main modular brain structures, called modular states (MS), that fluctuate over time during rest and task. We attempt to find the modular structures that share the same topology by quantifying the similarity between all the computed temporal partitions (Figure 1). The proposed framework simply takes as input a set of connectivity matrices, without making any constraint on how these matrices are computed. This means that it is independent of the choice of the connectivity method, the frequency range of interest, the temporal resolution of dynamic connectivity, and the regions of interest (ROIs). Connectivity matrices could be also extracted from different individuals, or different experimental conditions. The main purpose of the framework is to find the common modular states between the connectivity patterns interpreted. In the paper, we used simulated data to show the advantages of our method over other existing methods in terms of spatial and temporal accuracy. Then, using three different MEG/EEG datasets, recorded in rest and task from healthy subjects and/or patients, we demonstrate the feasibility of the method to reveal both time-varying functional connectivity and the corresponding spatial patterns, as compared with the state-of-the art findings. Overall, findings show that the algorithm was able to track the dynamics of brain networks in adapted timescale, offering a new way to explore the spatiotemporal organization of brain function.

## MATERIALS AND METHODS

We have developed two versions of the algorithm: (a) “categorical,” where we aim to find the main modular structures over time, with no interest in their sequential order; and (b) “consecutive,” where the objective is to find the modular structures in a successive way. The two versions are described hereafter.

### Categorical Version

The categorical version includes three main steps:

(1)  Decompose each temporal network into modules (i.e., clusters of nodes that are internally strongly connected, but externally weakly connected; Figure 1A). To do that, different modularity algorithms were proposed in the literature (Blondel, Guillaume, Lambiotte, & Lefebvre, 2008; Duch & Arenas, 2005; Girvan & Newman, 2002; Guimerà & Amaral, 2005).
  In our study, we adopted the consensus modularity approach that was previously used in many studies (Bassett et al., 2013; Kabbara et al., 2017): Given an ensemble of partitions acquired from the Newman algorithm (Girvan & Newman, 2002) and Louvain algorithm (Blondel et al., 2008) repeated for 200 runs, an association matrix is obtained. This results in an *N* × *N* matrix (*N* is the number of nodes), and an element *A*_*i*,*j*_ represents the number of times the nodes *i* and *j* are assigned to the same module across all runs and algorithms. The association matrix is then compared with a null model association matrix generated from a permutation of the original partitions, and only the significant values are retained (Bassett et al., 2013). To ultimately obtain consensus communities, we reclustered the association matrix using the Louvain algorithm.
(2)  Assess the similarity between the temporal modular structures (Figure 1B). In this context, several methods have been suggested to compare community structures (Traud, Kelsic, Mucha, & Porter, 2008). Here, we focused on the pair-counting method, which defines a similarity score by counting each pair of nodes drawn from the *N* nodes of a network according to whether the pair falls in the same or in different groups in each partition (Traud et al., 2008). We considered the z-score of Rand coefficient, bounded between 0 (no similar pair placements) and 1 (identical partitions). This yields a *T* × *T* similarity matrix, where *T* is the number of time windows.
(3)  Cluster the similarity matrix into “categorical” modular states (MS) using the consensus modularity method (Figure 1C). This step combines similar temporal modular structures in the same community. Hence, the association matrix of each “categorical” community is computed using the modular affiliations of its corresponding networks.

### Consecutive Version

**Figure 2.**
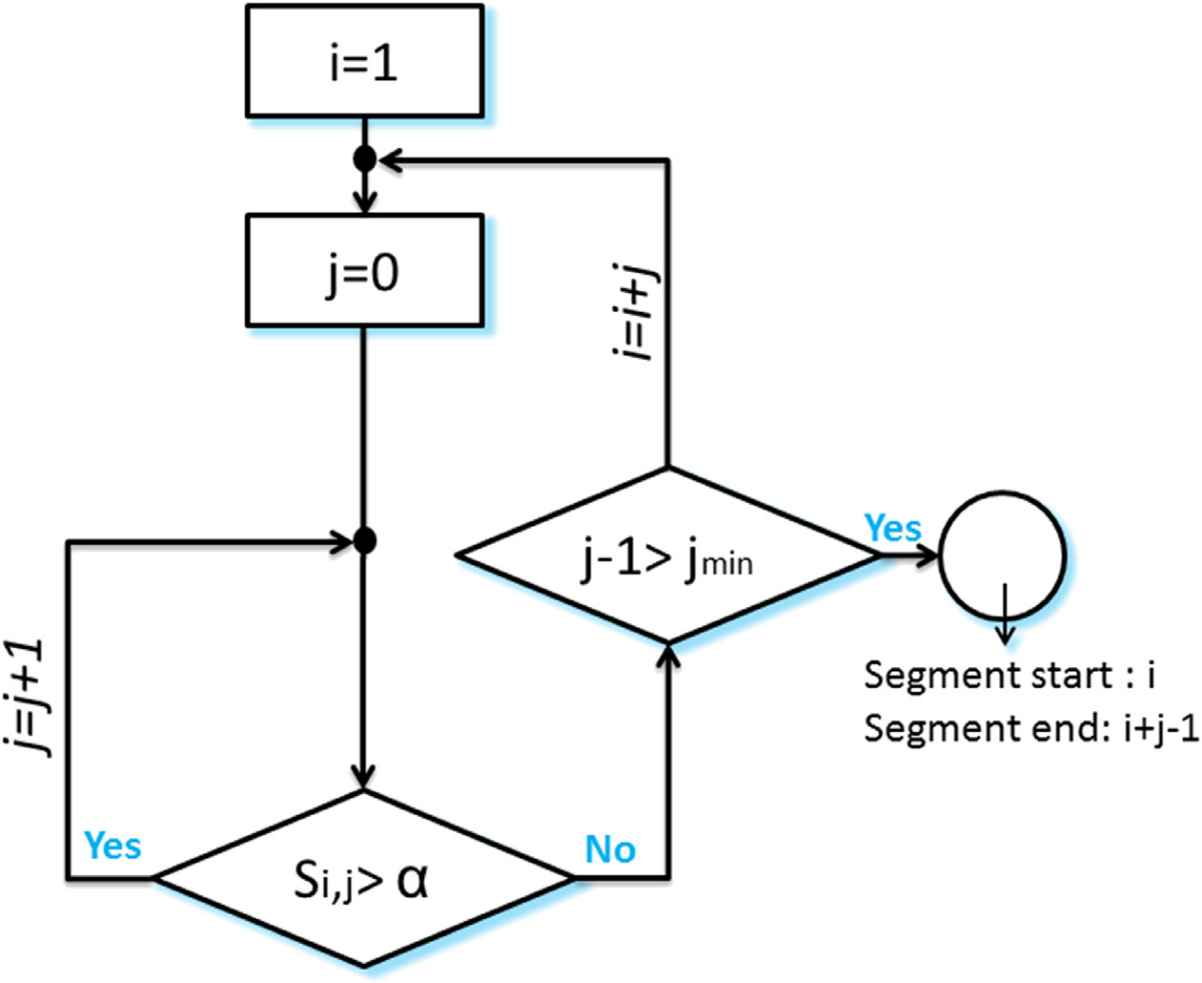
Flowchart of the segmentation algorithm.

The difference between the two versions of the algorithm is essentially in the third step, in which the final communities were defined. Particularly, the similarity matrix is segmented in a sequential way using the following steps:

(1)  Threshold the similarity matrix using an automatic thresholding algorithm described in Genovese, Lazar, and Nichols (2002). Briefly the matrix was converted into a *p* value map that is then thresholded based on the false discovery rate (FDR) controlling.
(2)  Apply a median filter in order to get a smoother presentation of the similarity matrix.
(3)  Segment the matrix in a sequential way following the algorithm illustrated in the flowchart of Figure 2. In brief, the method groups similar consecutive modular structures. As these modular structures show high similarity values with each other, the algorithm detects the squares located around the diagonal of the similarity matrix. As presented in Figure 2, the condition for which two consecutive structures are associated with the same state is the following:
  $$S_{i,j}>a,$$
  where *S*_*i*,*j*_ = $\sum_{k=0}^{j}$ *a*_*i*+*j*,*i*+*k*_ + $\sum_{k=0}^{j}$ *a*_*i*+*k*,*i*+*j*_ − *a*_*i*+*j*,*i*+*j*_; *i*, *j* ∈ [1, *T*], where *a*_*l*,*m*_ denotes the similarity value between the modular structure corresponding to the time window *l* and that corresponding to the time window *m*.

*a* is the “accuracy parameter,” strictly bounded between 0 and 1. It regulates the temporal-spatial accuracy of detected modular states. We recommend choosing an adaptive value of *a*. In this paper, *a* equals the average of the similarity matrix. A segment is considered to be relevant if the number of included time windows is greater than *j*_*min*_ (the minimal size allowed for a segment).

**Figure 3.**
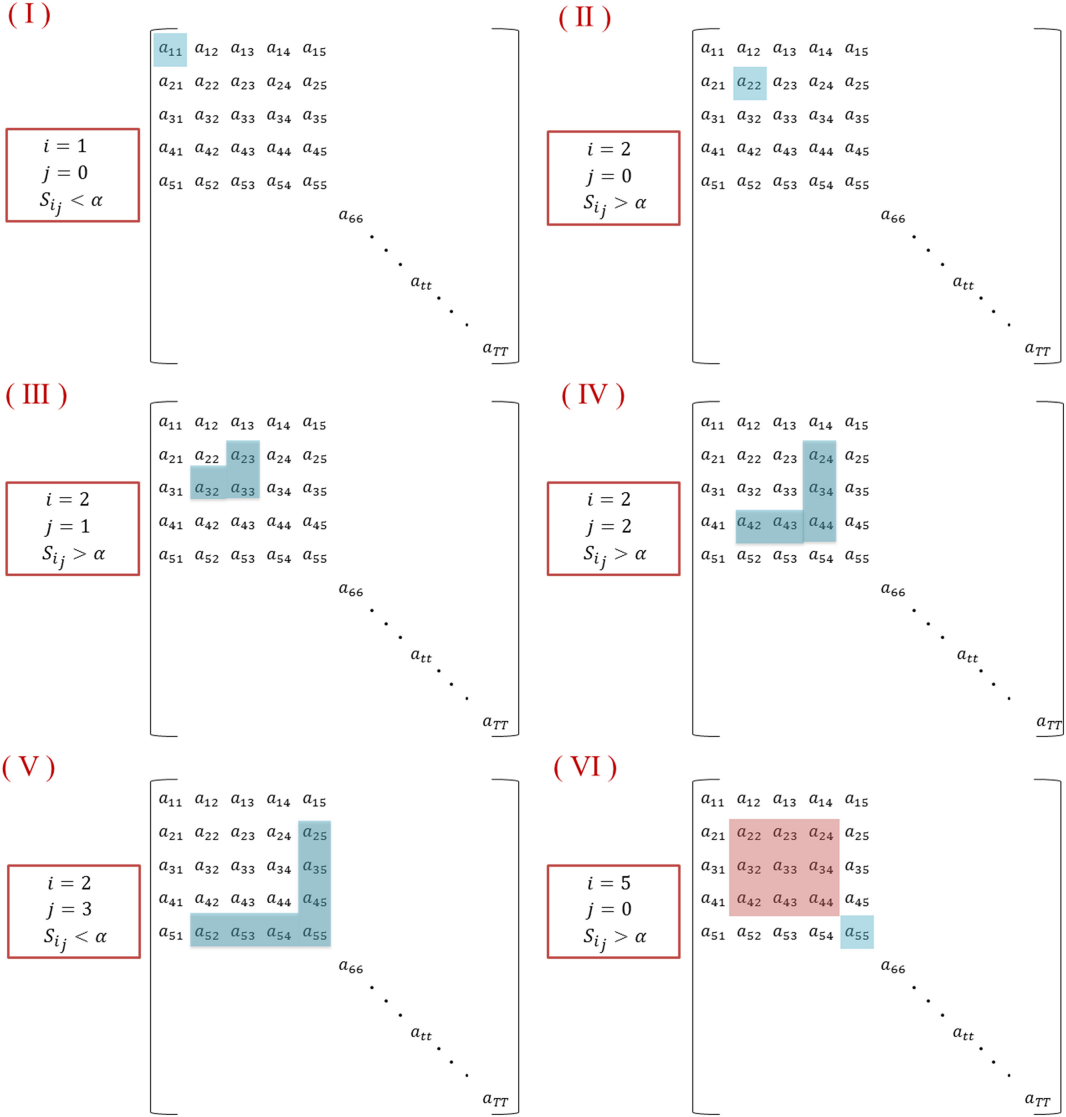
An illustrative example describing the segmentation algorithm.

The algorithm is illustrated in Figure 3. (I): Starting with*i* = 1, *j* = 0; and considering that *a*_11_ is lower than *a*, we obtained *S*_1,0_ = *a*_11_ < *a*, and the algorithm moves to the next time window *i* = 2. (II): As *S*_2,0_ = *a*_22_ is greater than *a*, *j* is incremented by 1. (III): Having *S*_2,1_ = $\frac{a_{23}+a_{23}+a_{33}}{3}$ > *a*, the second and the third time windows are associated with the segment. (IV): The algorithm succeeds to add also the fourth time window as *S*_2,2_ = $\frac{a_{24}+a_{42}+a_{34}+a_{43}{+a}_{44}}{5}$ > *a*. (V): Then, for *j* = 3, we obtain *S*_2,3_ = $\frac{a_{25}+a_{35}+a_{45}+a_{55}{+a}_{52}+a_{53}{+a}_{54}}{7}$ < *a*. This means that the fifth time window differs from the previous windows in its modular structure. Afterwards, the algorithm moves toward finding another segment by incrementing *i* and repeating the process (IV).

For each detected segment, the modular structure is obtained after computing the association matrix of the corresponding time windows modular affiliations.

The Matlab code developed to apply the algorithm in the two versions is publicly available (Kabbara et al., 2019).

### Simulated Data

**Figure 4.**
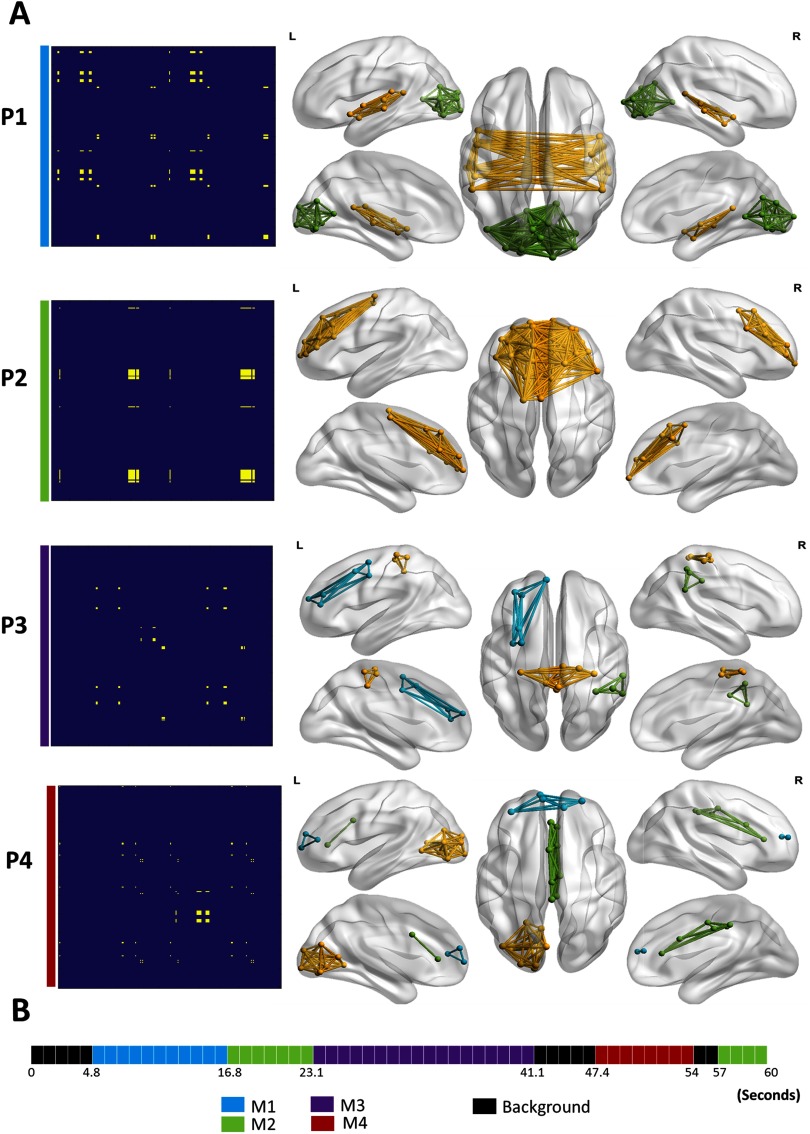
The simulation scenario. (A) Left: The adjacency matrix *P*_*j*_ of the constructed networks. Right: The 3-D cortical presentation of the modular structures of the simulated networks. (B) The time axis showing the beginning and the end of each network *M*_*j*_ (which was generated by combining *P*_*j*_ to uncorrelated noise).

We simulated the adjacency tensor data following the methodology applied in O’Neill, Tewarie, Colclough, et al. (2017). Briefly, four *N* × *N* adjacency matrices *P*_*j*_ were constructed, where *j* ∈ [1, 4] and *N* is the number of ROIs. We used an anatomical atlas of 221 ROIs with the mean of the Desikan-Killiany atlas (Desikan et al., 2006) subdivided by Hagmann et al. (2008), yielding to *N* = 221. The adjacency matrices and the 3-D visualization of the networks are presented in Figure 4A. Following this step, the time evolution of dynamic connectivity in each network is given by the following:

$$M_{j}(t)=a.f_{1j}(t)+b.f_{2j}(t);$$

*f*_1*j*_(*t*) is the modulation function, which was represented by the Hanning window of unit amplitude; *f*_2*j*_(*t*) represents uncorrelated Gaussian noise added to the simulated time courses; and *a* and *b* are scalar values set to 0.45 and 0.15 as in O’Neill, Tewarie, Colclough, et al. (2017).

In our study, *M*_*j*_(*t*) is sampled at $3.\bar{3}$ Hz (to obtain a sliding window of 0.3 s as in real data). The onset as well as the duration of each module structure is illustrated in Figure 4B. We then combined the four network matrices in order to generate a single adjacency matrix at each time point *t* over a time course spanning 60 s. As a final step, we added a random Gaussian noise to the adjacency tensor, and the standard deviation of the noise was allowed to vary between 0.2 and 0.5.

### Validation

On simulated data, we evaluated the performance of the algorithm by computing the similarity between the reconstructed and the simulated (reference) networks, taking into account both spatial and temporal similarities. The spatial similarity is given by the z-score of Rand coefficient between the simulated and the constructed modular structures, while the temporal similarity represents the rate of the correct affiliation of time windows.

In addition, we have compared our algorithm with three methods originally developed to identify the brain states without looking at their modular structures. The three methods are K-means clustering as applied by Allen et al. (2014), independent component analysis as suggested by O’Neill, Tewarie, Colclough, et al. (2017), and the consensus clustering algorithm proposed by Rasero et al. (2017). For each method, we followed the same pipeline adapted by the authors. To ultimately obtain the modular brain states, we preceded the analysis by decomposing each connectivity state to modules using the consensus modularity approach as detailed in the Categorial Version section.

### Real Data

In order to track the brain network dynamics effectively, we particularly tested our method using electro- and magnetoencephalography (EEG/MEG), which allow the tracking of brain dynamics on a subsecond timescale, a resolution not reachable using other techniques such as functional magnetic resonance imaging (fMRI; Cohen, 1972; Nunez & Srinivasan, 2007; Penfield & Jasper, 1954).

Thus, three M/EEG datasets were analyzed. Before applying the algorithm, we reconstucted the functional brain networks at the level of the cortex using an emerging technique called EEG source connectivity (Chavez et al., 2013; De Vico Fallani et al., 2008; De Vico Fallani, Latora, & Chavez, 2017; De Vico Fallani, Richiardi, Chavez, & Achard, 2014; Hassan, Shamas, Khalil, El Falou, & Wendling, 2015; Hassan & Wendling, 2018; Kabbara et al., 2017; Lai, Demuru, Hillebrand, & Fraschini, 2018; Schoffelen & Gross, 2009). In order to remove any bias from our analysis, we have generated the dynamic functional connectivity matrices according to how they were computed in the three previous studies that interpreted the data (Hassan, Benquet, et al., 2015; Hassan et al., 2017; O’Neill, Tewarie, Colclough, et al., 2017) The aim of this work is to test whether the modular states generated by our algorithm match the findings obtained in the literature.

#### Dataset 1: Self-paced motor task for healthy participants (MEG data).

Previously used in O’Neill et al. (2015), O’Neill, Tewarie, Colclough, et al. (2017), and Vidaurre et al. (2016), this dataset includes 15 participants (9 male, 6 female) asked to press a button using the index finger of their dominant hand, once every 30 s. Using a 275-channel CTF MEG system (MISL; Coquitlam, BC, Canada), MEG data were recorded at a sampling rate of 600 Hz. MEG data were coregistered with a template MRI. The cortex was parcellated using the Desikan-Killiany atlas (68 regions; Desikan et al., 2006). The preprocessing, the source reconstruction, and the dynamic functional connectivity computations were performed similarly to those in O’Neill, Tewarie, Colclough, et al. (2017). Briefly, the preprocessing comprises the exclusion of trials (t = −12s → 12s) contaminated by noise. Then, source time courses were reconstructed using a beamforming approach (please refer to O’Neill, Tewarie, Colclough, et al., 2017, for more details). Afterwards, the regional time series were symmetrically orthogonalized following the method proposed in Colclough, Brookes, Smith, and Woolrich (2015) to remove the effects of “signal leakage.” The amplitude envelopes of the time courses were obtained using a Hilbert transform. Finally, the dynamic connectivity was estimated by the Pearson correlation measure using a sliding window approach of 6 s of length. The sliding window was shifted by 0.5 s over time. The number of connectivity matrices obtained for each trial was then 49. A statistical threshold (FDR) approach was used to threshold the matrices (Genovese, Lazar, & Nichols, 2002). This yields to a set of connectivity matrices that are thresholded and weighted. Then, the consecutive scheme of the proposed method was tested.

#### Dataset 2: Picture naming task for healthy participants (dense-EEG data).

Twenty-one right-handed healthy subjects (11 women and 10 men) with no neurological disease participated in this study. In a session of about 8 min, each participant was asked to name 148 displayed pictures on a screen using E-Prime 2.0 software (Psychology Software Tools, Pittsburgh, PA; Schneider, Eschman, & Zuccolotto, 2002). Oral responses were recorded to set the voice onset time. This study was approved by the National Ethics Committee for the Protection of Persons (CPP), ConneXion study, agreement number 2012-A01227-36, and promoter, Rennes University Hospital. All participants provide their written informed consent to participate in this study. A typical trial started with the appearance of an image during 3 s followed by a jittered interstimulus interval of 2 or 3 s randomly. Errors in naming were discarded from the analysis. A total of 2,926 on 3,108 events were considered.

Dense-EEG data were recorded using a system of 256 electrodes (EGI, Electrical Geodesic, Inc.). EEGs were collected at 1 kHz sampling frequency and bandpass filtered between 3 and 45 Hz. The preprocessing and the computation of the functional connectivity followed the same pipeline applied in Hassan, Benquet, et al. (2015). In brief, each trial (*t* = 0 → 600 ms) was visually inspected, and epochs contaminated by eye blinks, muscle movements, or other noise sources were rejected. As described in the previous study (Hassan, Benquet, et al., 2015), the source connectivity method was performed using the wMNE/PLV (phase locking value) combination, and the dynamic functional connectivity was computed at each millisecond. Authors also used the Destrieux atlas subdivided into 959 regions (Hassan, Benquet, et al., 2015). To remove spurious connections from the dynamic connectivity matrices, we have adopted a statistical threshold based on FDR (Genovese et al., 2002). Finally, a weighted and thresholded connectivity tensor of dimension 959 × 959 × 600 was obtained and analyzed using the consecutive scheme of our algorithm.

#### Dataset 3: Resting state in Parkinson’s disease patients (dense-EEG data).

This dataset includes 124 patients with idiopathic Parkinson’s disease defined according to the UK Brain Bank criteria for idiopathic Parkinson’s disease (Gibb & Lees, 1988). These patients were separated into three groups: (G1) cognitively intact patients (*N* = 63); (G2) patients with mild cognitive deficits (*N* = 46); and (G3) patients with severe cognitive impairment (*N* = 15). All participants gave their informed consent to participate in the study, which had been approved by the local institutional review boards (CPP Nord-Ouest IV, 2012-A 01317-36, ClinicalTrials.gov Identifier: NCT01792843). Dense-EEG were recorded with a cap (Waveguard, ANT software BV, Enschede, Netherlands) with 122 scalp electrodes distributed according to the international system 10-05 (Oostenveld & Praamstra, 2001). Electrode impedance was kept below 10 kΩ. Patients were asked to relax without performing any task. Signals were sampled at 512 Hz and bandpass filtered between 0.1 and 45 Hz.

The data were preprocessed according to Hassan et al. (2017) dealing with the same dataset. Briefly, EOG artifact detection and correction was applied following the method developed in Gratton, Coles, and Donchin (1983). Afterwards, epochs with voltage fluctuation between 90 *μ*V and −90 *μ*V were kept. For each participant, two artifact-free epochs of 40-s lengths were selected. This epoch length was used previously and considered as a good compromise between the needed temporal resolution and the reproducibility of the results in resting state (Kabbara et al., 2017).

To compute the dynamic functional connectivity, the steps adopted here are the same used in many previous studies (Hassan et al., 2017; Kabbara et al., 2018, 2017). First, EEG data were coregistered with a template MRI through identification of the same anatomical landmarks (left and right preauricular points and nasion). Second, the lead field matrix was computed for a cortical mesh with 15,000 vertices using the OpenMEEG package (Gramfort, Papadopoulo, Olivi, & Clerc, 2010) available in Brainstorm. The noise covariance was estimated using a 1-min resting segment. After that, the time series of EEG sources were estimated using the wMNE algorithm where the regularization parameter was set according to the signal to noise ratio (*λ* = 0.1 in our analysis). An atlas-based segmentation approach was used to project EEGs onto an anatomical framework consisting of 68 cortical regions identified by means of the Desikan-Killiany atlas (Desikan et al., 2006). The dynamic functional connectivity was then computed using a sliding window over which PLV was calculated (Lachaux, Rodriguez, Martinerie, & Varela, 1999). In the previous study (Hassan et al., 2017), the disruptions of the functional connectivity were found in the alpha 2 band (10–13 Hz). For this reason, we considered the same frequency band in our analysis. To obtain a sufficient number of cycles at the given frequency band, we chose the smallest window length that is equal to $\frac{\text{6}}{\text{central frequency}}$, as recommended in Lachaux et al. (1999). This yields a sliding window of 0.52 s. We then adopted a proportional threshold of 10% to maintain only the top 10% of correlation values of the connectivity matrix. These steps produce, for each epoch, a weighted thresholded connectivity tensor of dimension *N* × *N* × *T*, where *N* is the number of ROIs (68 regions) and *T* is the number of time windows (77 time windows). This tensor is formally equivalent to dynamic functional connectivity matrices, and it was analyzed using the categorical version of the proposed algorithm.

## RESULTS

Having the set of connectivity matrices, we first applied a community detection algorithm (Louvain method) to decompose each network into modules (i.e., clusters of nodes that are internally strongly connected, but externally weakly connected). The similarity between the temporal modular structures was calculated (Figure 1B). Finally, the modular states (MS) were obtained by applying a community detection algorithm to the similarity matrix (Figure 1C).

We propose two different frameworks: (a) “categorical,” where the objective is to find the main modular structures, without any interest in their sequential order; and (b) “consecutive,” where the objective is to find the modular structures in a successive way.

### Validation on Simulated Data

**Figure 5.**
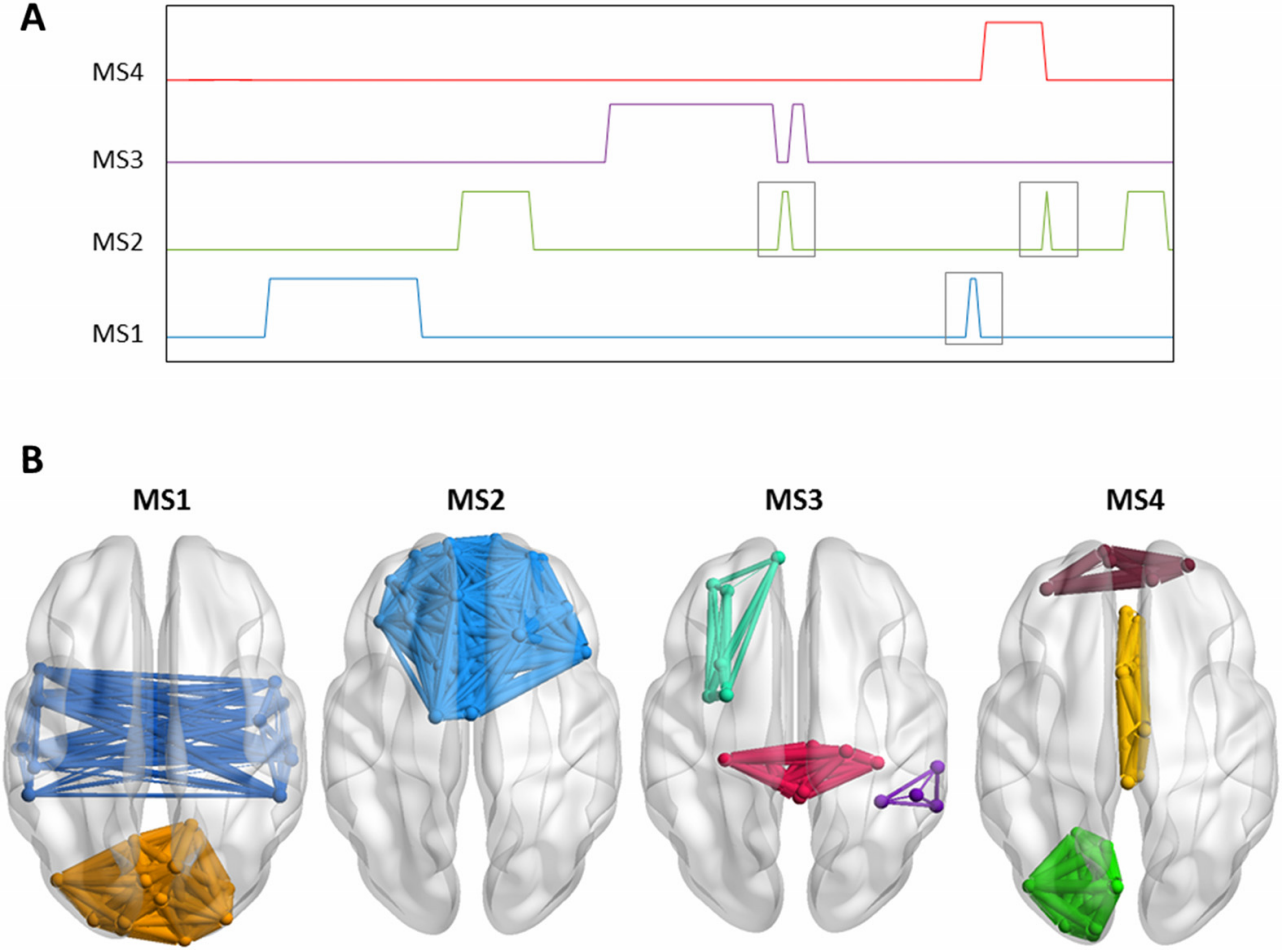
Results of the categorical method applied on simulated data. (A) The time course of the four modular structures reconstructed. The gray square indicates false time window detection. (B) The 3-D representation of the four modular structures’ states.

Figure 5 shows the results of the categorical method applied on the dynamic networks generated by the simulation scenario (*STD*_*noise*_ = 0.2). Four modular states were obtained: MS1, MS2, MS3, and MS4. Figure 5A illustrates the modular states’ time courses, showing the most likely state at each time window, while Figure 5B shows the 3-D representation of the MS. Qualitatively, the four simulated modular structures have been successfully reconstructed. However, one time window that actually belongs to the background (i.e., random) has been wrongly affiliated with MS2. Moreover, the MS3 state time course presented two false time window detections: one belongs to MS4, and the other belongs to the background. To quantitatively validate the obtained results, we compared the simulated structures (M1, M2, M3, and M4) with the reconstructed structures (MS1, MS2, MS3, and MS4) in terms of spatial and temporal similarities. The spatial similarities between the simulated and the reconstructed data are 0.99, 0.98, 0.99, and 0.98 for MS1, MS2, MS3, and MS4, respectively. The temporal similarities are 0.79, 0.83, 0.9, and 0.71 for MS1, MS2, MS3, and MS4, respectively. On average, the categorical method achieved spatial accuracy of 98.5% and a temporal accuracy of 80.8%.

**Figure 6.**
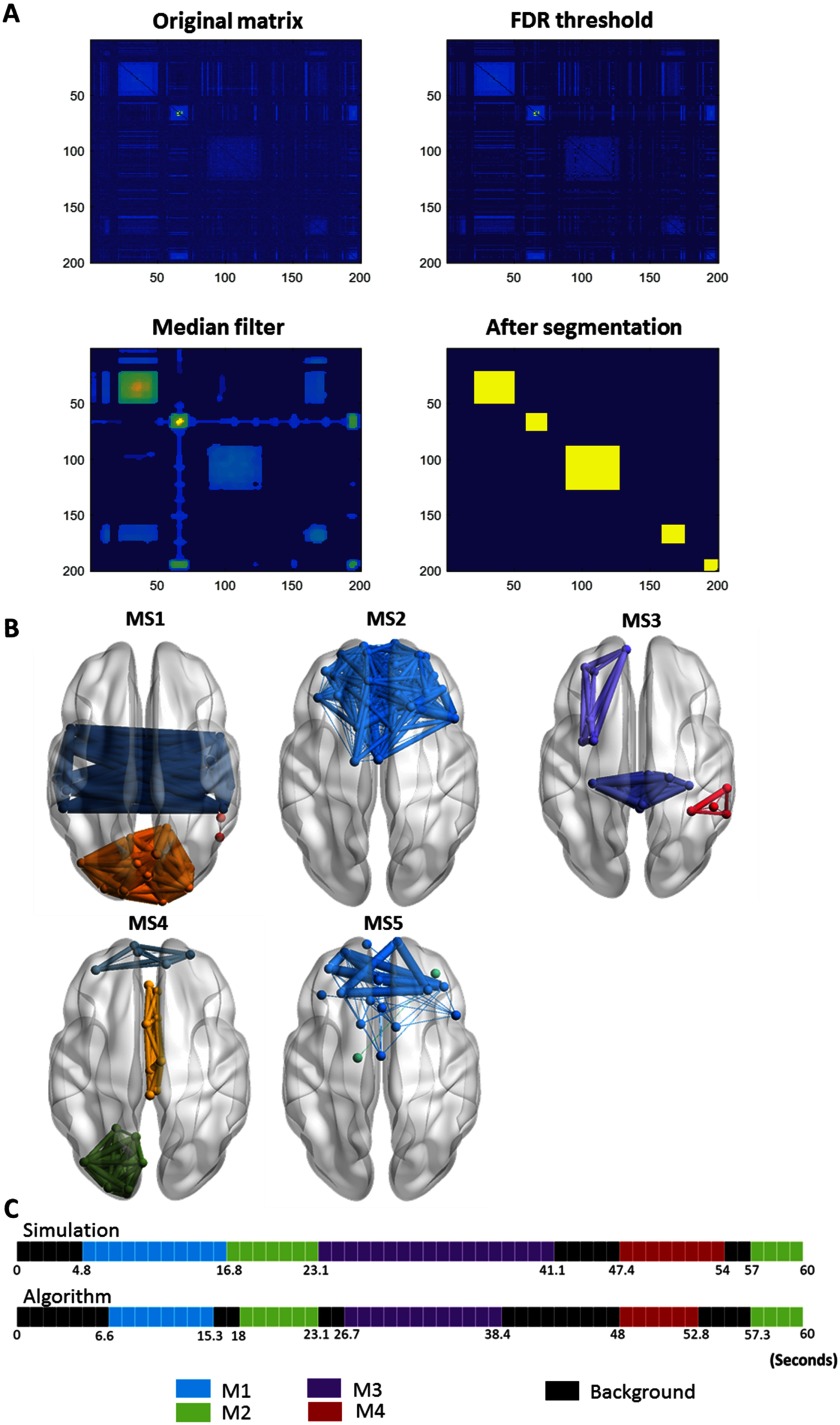
Results of the consecutive method applied on simulated data. (A) The results of the segmentation algorithm used to derive the consecutive modular structures from the similarity matrix by (1) thresholding the matrix using FDR, (2) applying a median filter on the thresholded matrix, and (3) extracting the most significant segments. (See the Methods section for more details about the consecutive algorithm steps.) (B) The 3-D representation of the five consecutive modular structures obtained. (C) The difference between the simulated time axis and the obtained time axis.

Using the consecutive method, the algorithm has segmented the similarity matrix yielding to the detection of five modular states (Figure 6A). Their 3-D representations are shown in Figure 6B. One can remark that MS1 (spatial similarity = 0.94; temporal similarity = 0.88), MS2 (spatial similarity = 0.99; temporal similarity = 0.94), MS3 (spatial similarity = 0.97; temporal similarity = 0.77), MS4 (spatial similarity = 1; temporal similarity = 0.88), and MS5 (spatial similarity = 0.95; temporal similarity = 1) matched, temporally and spatially, the simulated networks generated at the corresponding time windows. In addition, one can remark that the algorithm hasn’t produced false positive results. Globally, the assessed spatial and temporal accuracies are 94% and 83%, respectively.

Results corresponding to *STD*_*noise*_ = 0.35; *STD*_*noise*_ = 0.5 are illustrated in the Supporting Information. In brief, results show that using the categorical algorithm, the spatial characterizations of the four modular states were successfully detected. However, the state time course of MS3 failed to detect the second corresponding segment (Figures 1 and 2 in the Supporting Information). Using the consecutive algorithm, the five MSs were temporally detected (Figures 3 and 4 in the Supporting Information).

**Figure 7.**
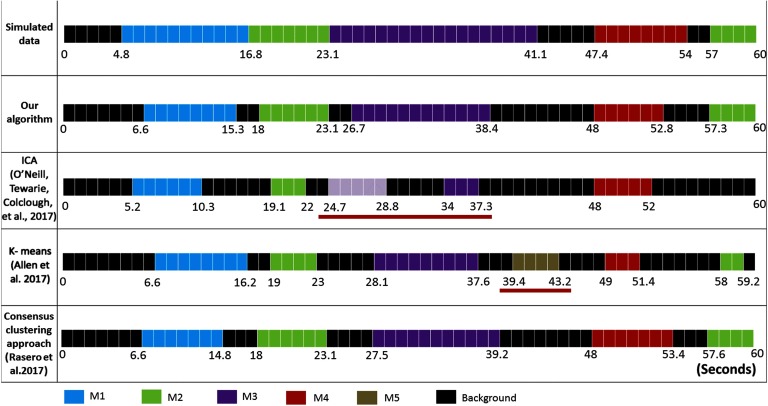
The difference between the simulated time axis and the obtained time axis using the different algorithms.

The difference between the simulated time axis and the obtained time axis using other different algorithms is presented in Figure 7. Using ICA (O’Neill, Tewarie, Colclough, et al., 2017), five independent components (ICs) were detected: IC1, IC2, and IC5 correspond, respectively, to M1 (spatial similarity = 0.96; temporal similarity = 0.43), M2 (spatial similarity = 0.85; temporal similarity = 0.60), and M4 (spatial similarity = 0.86; temporal similarity = 0.71). However, M3 (spatial similarity = 0.94; temporal similarity = 0.42) was reflected by two separated components: IC3 and IC4. One can realize also that the ICA algorithm failed to detect M2 spanning between 57 ms and 60 ms. Using K-means clustering (Allen et al., 2014), six brain states (BSs) were generated. BS1, BS2, BS3, BS5, and BS6 correspond, respectively, to M1 (spatial similarity = 0.96; temporal similarity = 0.8), M2 (spatial similarity = 0.94; temporal similarity = 0.77), M3 (spatial similarity = 0.97; temporal similarity = 0.53), M4 (spatial similarity = 1; temporal similarity = 0.61), and M5 (spatial similarity = 0.99; temporal similarity = 0.55). While the five simulated modular states were reconstructed, a false time window detection was captured (BS4). Regarding the consensus clustering algorithm proposed by Rasero et al. (2017), the algorithm succeeded to detect the five simulated states. Tables 1 and 2 report the temporal and spatial similarities between the simulated MSs and those obtained using the different algorithms. Overall, results show that our algorithm reached the highest temporal detection accuracy (0.83) compared with ICA (0.43), K-means clustering (0.58), and the consensus clustering (0.79). Spatially, our algorithm procured a good spatial similarity (0.97), outperforming ICA (0.72) and the consensus clustering algorithms (0.91).

**Table 1.** Temporal similarities between the simulated MSs and those obtained using the different algorithms

**Algorithm**	**MS1**	**MS2**	**MS3**	**MS4**	**MS5**	**False positive rate**	**Average temporal similarity**
**Our algorithm**	0.73	0.98	0.67	0.86	0.93	0	0.83
**ICA (O’Neill, Tewarie, Colclough, et al., 2017)**	0.43	0.6	0.41	0.71	0	0	0.43
**K-means (Allen et al., 2017)**	0.8	0.77	0.53	0.61	0.55	0.07	0.58
**Consensus clustering approach (Rasero et al., 2017)**	0.68	0.98	0.65	0.79	0.85	0	0.79

**Table 2.** Spatial similarities between the simulated MSs and those obtained using the different algorithms

**Algorithm**	**MS1**	**MS2**	**MS3**	**MS4**	**MS5**	**Average spatial similarity**
**Our algorithm**	0.94	0.99	0.97	1	0.95	0.97
**ICA (O’Neill, Tewarie, Colclough, et al., 2017)**	0.96	0.85	0.94	0.86	0	0.72
**K-means (Allen et al., 2017)**	0.96	0.94	0.97	1	0.99	0.99
**Consensus clustering approach (Rasero et al., 2017)**	0.92	0.96	0.89	0.91	0.89	0.91

### Real Data

#### Dataset 1: Self-paced motor task for healthy participants (MEG data).

**Figure 8.**
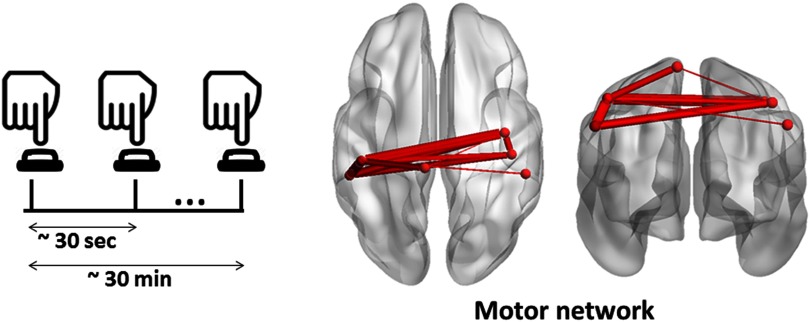
The MS of the MEG motor task obtained using the consecutive method.

As the considered dataset is collected during a task-related paradigm, our objective was to follow the spatiotemporal organization of the dynamic brain networks during time (from the stimulus onset to the reaction time). Thus, we applied the consecutive algorithm to track the MSs in a successive way. The input of the algorithm is the tensor of dynamic connectivity matrices averaged over all trials and subjects. The same dataset and methods were previously used in O’Neill et al. (2015), O’Neill, Tewarie, Colclough, et al. (2017), and Vidaurre et al. (2016). The algorithm results in one significant MS found between −0.5 s and 1.5 s (Figure 8). As illustrated, this module implicates the sensory motor area, the post- and precentral regions of both hemispheres.

#### Dataset 2: Picture naming task for healthy participants (dense-EEG data).

The objective of using this dataset was to track the fast space/time dynamics of functional brain networks at subsecond timescale from the onset (presentation of the visual stimuli) to the reaction time (articulation). Hence, the consecutive version was applied on the dynamic connectivity matrices averaged over subjects.

**Figure 9.**
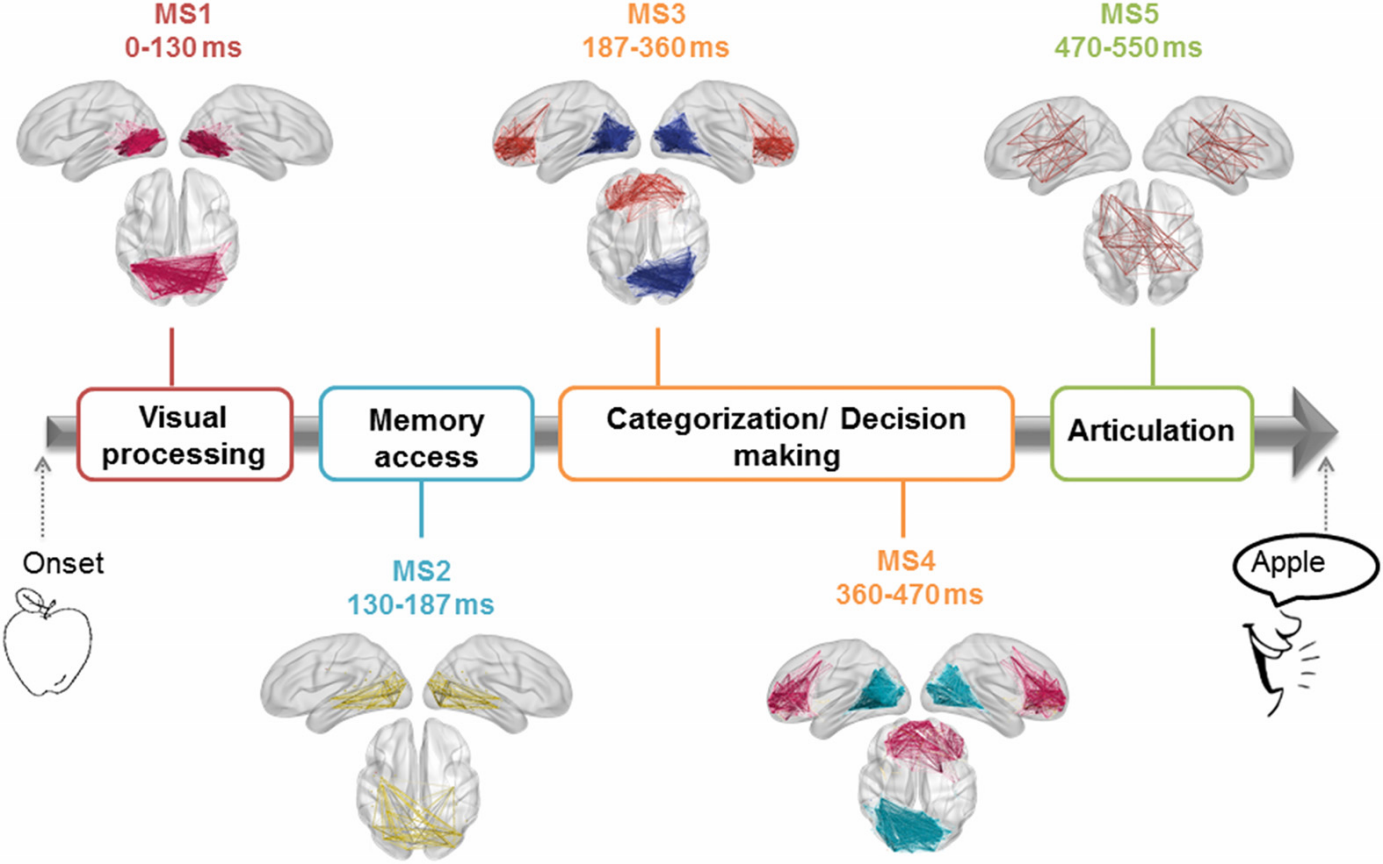
The sequential MSs of the EEG picture naming task obtained using the consecutive method and their corresponding cognitive functions.

Figure 9 shows the results, revealing that the cognitive process can be divided into five modular structures: The first MS corresponds to the time period ranging from the stimulus onset to 130 ms and presents one module located mainly in the occipital region. The second MS is observed between 131 and 187 ms, and involves one module showing occipitotemporal connections. The third MS is identified between 188 and 360 ms, and illustrates a module located in the occipitotemporal region, and another module located in the frontocentral region. This structure was then followed by a fourth MS, found over the period 361–470 ms. MS4 was very similar to the previous MS but with additional frontocentral connections. The last MS is observed between 471 and 500 ms, and it shows a module connecting the frontal, central, and temporal regions. It is worth noting that these MSs denote the transitions from the visual processing and recognition to the semantic processing and categorization to the preparation of the articulation process (Hassan, Benquet, et al., 2015; VanRullen & Thorpe, 2001).

#### Dataset 3: Resting state in Parkinson’s disease patients (dense-EEG data).

**Figure 10.**
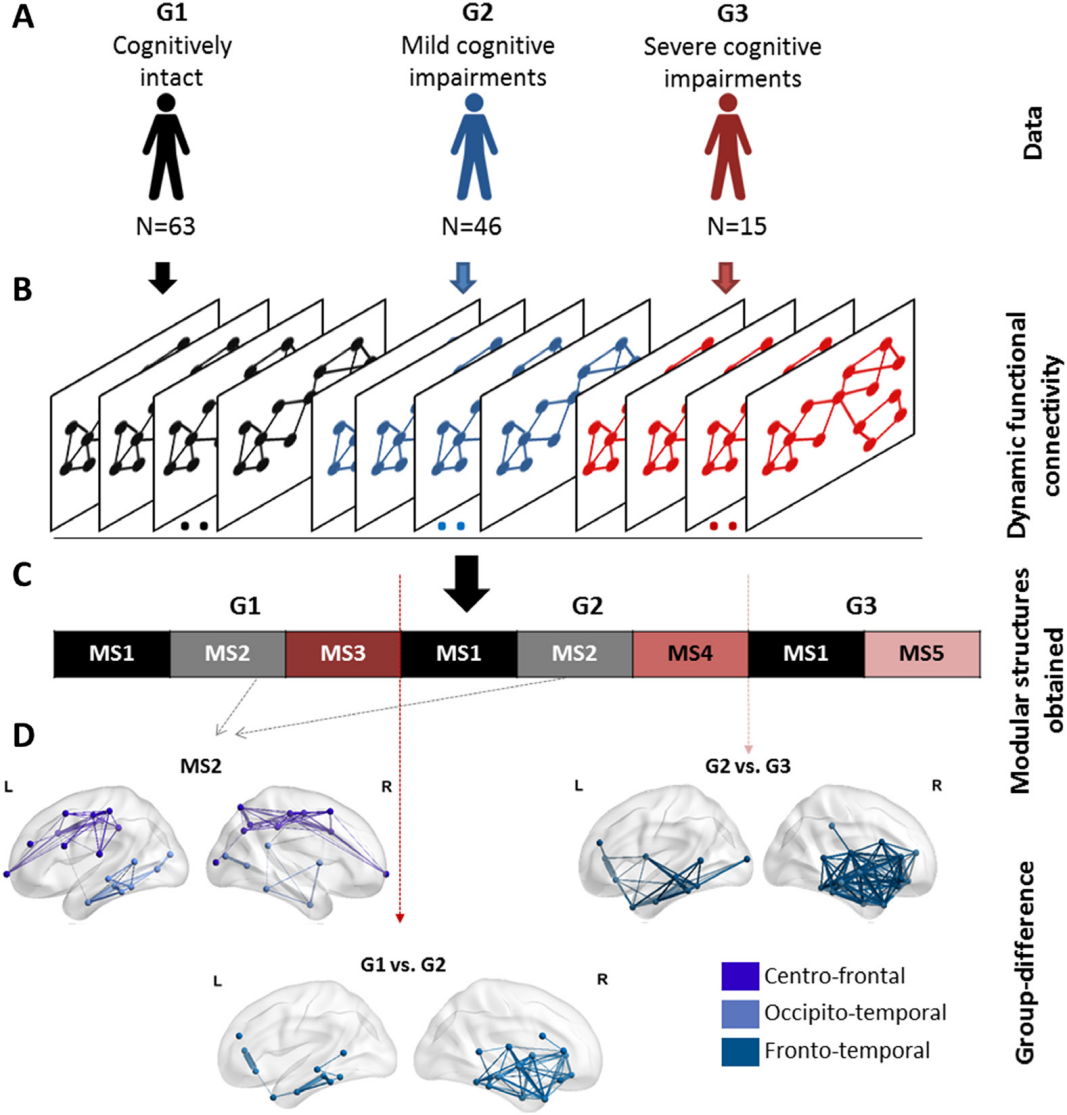
The analysis pipeline and the results of the categorical method applied on the Parkinson’s disease EEG dataset. (A) The dataset composed of 124 patients partitioned into three groups: (G1) cognitively intact patients (*N* = 63), (G2) patients with mild cognitive deficits (*N* = 46), and (G3) patients with severe cognitive impairment (*N* = 15). (B) The functional dynamic connectivity matrices of the three groups concatenated over time. (C) The five modular structures obtained after applying the categorical algorithm on the concatenated tensor. (D) The modular differences between G1 and G2, G1 and G3, and G2 and G3.

Our objective here was to identify the modular structures that are common between G1, G2, and G4, and those that are specific to each group. As the sequential order of MSs is not intended, we applied the categorical version of the proposed algorithm. The latter takes as input the concatenation of the dynamic connectivity matrices of the three groups. This will form a single data tensor of dimension *N* × *N* × *T*, where *N* is the number of ROIs and *T* is equal to the number of time windows × the number of patients (Figure 10). The algorithm was then applied to validate the usefulness of the categorical version in detecting the modular alterations between G1, G2, and G3.

Results are illustrated in Figure 10. Five modular structures were identified (MS1, MS2, MS3, MS4, and MS5). Three MSs were found for G1 and G2. However, the number of MSs decreased from three to two MSs in G3. Results revealed that MS1 was found to be present in the three groups, while MS2 was present only in G1 and G2. The modular structure MS2 (absent in G3) is illustrated in Figure 10 and includes two modules involving mainly frontocentral and occipitotemporal connections. The difference between G1 and G2 was reflected by the absence of the structure MS3 replaced by the structure MS4 in G2. Results in Figure 10 show that the difference concerns mainly the frontotemporal connections. The difference between G2 and G3 was reflected by the absence of the structure MS4 from G2 and the presence of the structure MS5 in G3. Figure 10 shows that the functional disruptions between G2 and G3 are mainly frontotemporal connections. It is worth noting that the frontotemporal disruptions were widely reported in mild cognitive impairments (Beyer, Janvin, Larsen, & Aarsland, 2007; de Haan et al., 2012; Song et al., 2011; Zhang et al., 2009), while the central disruptions are widely observed in severe cognitive impairments (Hassan et al., 2017) and dementia (Song et al., 2011).

## DISCUSSION

In this paper, we have developed a novel framework to explore the fast reconfiguration of the functional brain networks during rest and task. This new method can be used to track the sequential evolution of brain modules during a task-directed paradigm or to identify the modular brain states that arise at rest. The simulation-based analysis showed the ability of the method to “reestimate” the modular network structures over time. Compared with other methods, our proposed algorithm reached the highest temporal accuracy detection and a very good spatial accuracy.

The new framework was validated on three different EEG/MEG datasets: (a) MEG data recorded from 15 healthy subjects during a self-paced motor task, (b) dense-EEG data recorded from 21 healthy subjects during a picture naming task, and (c) dense-EEG data recorded at rest from 124 Parkinson’s disease patients with different cognitive phenotypes. Results show that our method can track the fast modular states of the human brain network at a subsecond timescale, and also highlight its potential clinical applications, such as the detection of the cognitive decline in Parkinson’s disease.

### “Categorical” and “Consecutive” Processing Schemes

The two processing schemes proposed here are both derived from the similarity matrix between the temporal modules (Steps 1 to 3 in the Methods section). However, each version highlights a specific characterization of the modular structures, which can then be exploited depending on the application (time/condition dependent). In particular, the results of the categorical algorithm on the simulated data reveal high spatial resolution and relatively low temporal resolution compared with those obtained using the consecutive algorithm. The low temporal resolution of the categorical version is reflected by the false (Figure 5) as well as the missed time windows detection (Figures 1 and Figure 2 in the Supporting Information). In contrast, these time windows were correctly detected by the consecutive version despite their short length (Figure 6, and Supporting Information Figures 3 and 4). Yet, the low spatial resolution of the consecutive version can be illustrated by MS5 (Figure 6, and Supporting Information Figures 3 and 4) that should represent M2 (Figure 4). This is probably due to the categorical version using the maximum number of available data points to generate their corresponding MS, while the consecutive version treats each temporal segment solely.

We suggest using the consecutive version where sequential order of MSs is interesting to investigate, such as the tracking of cognitive tasks. When the temporal aspects are not necessary, we would recommend the categorical version.

### Tracking of Fast Cognitive Functions

The brain dynamically reconfigures its functional network structure on subsecond temporal scales to guarantee efficient cognitive and behavioral functions (O’Neill, Tewarie, Vidaurre, et al., 2017). Tracking the spatiotemporal dynamics of large-scale networks over this short time duration is a challenging issue (Allen et al., 2014; Hutchison et al., 2013). In this paper, we aimed to examine how fast changes in the modular architecture shape information processing and distribution in (a) motor tasks and (b) picture naming tasks.

Concerning the self-paced motor task, it is a simple task where only motor areas are expected to be involved over time. Our results indeed showed that the motor module is clearly elucidated related to the tactile movement of the button press. The spatial and temporal features of the obtained module are very close to the significant component obtained by O’Neill, Tewarie, Colclough, et al. (2017) using the temporal ICA method.

The different MSs obtained in the EEG picture naming task are temporally and spatially analogous to the network brain states detected using other approaches such as K-means clustering by Hassan, Benquet, et al. (2015). In particular, the first MS representing the visual network is probably modulated by the visual processing and recognition processes (Thorpe, Fize, & Marlot, 1996; VanRullen & Thorpe, 2001). The second MS reflects the memory access reflected by the presence of the occipital-temporal connections (Martin & Chao, 2001). In other words, the brain tries to retrieve the information related to the picture illustrated from memory (Martin & Chao, 2001). In the third and the fourth MSs, we notice the implication of a separated frontal module. This module may be related to the object category recognition (tools vs. animals) and the decision-making process (Andersen & Cui, 2009; Clark & Manes, 2004; Rushworth, Noonan, Boorman, Walton, & Behrens, 2011). After making the decision, speech articulation and the naming process is prepared and started (Dronkers, 1996). This is reflected by the MS5 that combines the frontal, the motor, and the temporal brain areas.

### Modular Brain States and Cognitive Phenotypes in Parkinson’s Disease

Emerging evidence shows that Parkinson’s disease (PD) is associated with alteration in structural and functional brain networks (Fornito, Zalesky, & Breakspear, 2015). Hence, from a clinical perspective, the demand is high for a network-based technique to identify the pathological networks and to detect early cognitive decline in PD. Here, we used a dataset with a large number (*N* = 124) of PD patients categorized into three groups in terms of their cognitive performance: G1, cognitively intact patients; G2, patients with mild to moderate cognitive deficits; and G3, patients with severe cognitive deficits in all cognitive domains. See Dujardin et al. (2015), Hassan et al. (2017), and Lopes et al. (2017) for more information about this database.

The obtained MSs presented in Figure 10 show that while some MSs remain unchangeable during cognitive decline from G1 to G3, others are altered and replaced by new MSs. More specifically, the number of MSs detected in G3 has decreased compared with G1 and G2; MS3 in G1 was replaced by MS4 in G2, while MS4 in G2 was replaced by MS5 in G3. In addition, the alterations in G3 involve more distributed modules (central, frontotemporal) than the alterations occurring between G1 and G2 (frontotemporal modules) where the impairment still moderate.

Interestingly, the underlying modular differences between the MSs of groups are consistent with the previously reported studies that explored the network changes in PD (Beyer et al., 2007; Bosboom, Stoffers, Wolters, Stam, & Berendse, 2009; de Haan & Pijnenburg, 2009; Hassan et al., 2017; Song et al., 2011; Zhang et al., 2009). Particularly, the loss of frontotemporal connections in PD is supported by several EEG and MEG studies (Bosboom et al., 2009; Hassan et al., 2017; Zhang et al., 2009). Similarly, results of structural MRI studies reveal frontal and temporal atrophies in PD with mild cognitive impairment (Beyer et al., 2007; Song et al., 2011). Other functional (de Haan et al., 2012) and structural (Zhang et al., 2009) studies showed that Alzheimer’s disease networks are characterized by frontotemporal alterations. In addition, the brain regions involved in the modular alterations in G3 found in our study are in line with findings obtained by EEG edgewise analysis (Hassan et al., 2017), and by structural MRI studies showing widespread atrophy associated with PD patients’ related dementia (Song et al., 2011).

### Methodological Considerations

First, we used a template anatomical image generated from MRIs of healthy controls for EEG/MEG source functional connectivity analysis. The template-based method is common practice in the absence of individual anatomical images and was previously employed by multiple EEG and MEG source reconstruction studies, because of nonavailability of native MRIs (Hassan et al., 2017; Kabbara et al., 2018; Lopez et al., 2014). Furthermore, a recent study showed that there are few potential biases introduced during the use of a template MRI compared with individual MRI coregistration.

Second, the connectivity matrices in Dataset 3 (Parkinson’s disease analysis) were thresholded by keeping the highest 10% of the edge’s weights. According to previous studies, the 10% threshold provides an optimal trade-off between retaining the true connections and reducing spurious connections (Lord, Horn, Breakspear, & Walter, 2012). However, the consistency of results across a range of proportional thresholds would be interesting to consider. In contrast, a statistical threshold (FDR) approach was used in Dataset 1 (self-paced motor tasks) and Dataset 2 (picture naming); see Methods. The reason is that in Dataset 3, three groups were compared. The proportional threshold approach, compared with other threshold approaches, ensures equal density between the analyzed groups (van den Heuvel et al., 2017). Moreover, studies suggest that FDR controlling procedures are effective for the analysis of neuroimaging data in the absence of intergroup comparison (Bassett et al., 2013; Genovese et al., 2002; Patel & Bullmore, 2016). One should also note that other strategies and criteria could be used to objectively threshold the connectivity matrices, such as the minimum spanning tree metric (Tewarie, van Dellen, Hillebrand, & Stam, 2015) and the efficiency cost optimization (De Vico Fallani et al., 2017).

Third, according to the consecutive algorithm, it is noteworthy that two parameters should be adequately tuned to obtain relevant results: (a) the accuracy parameter *a*, and (b) the minimal length allowed for a state *j*_*min*_. In particular, the accuracy parameter *a* will dramatically affect the number and the temporal accuracy of detected states. Thus, it should be appropriately selected. A high value may eliminate strong and significant similarity values in a low-average matrix, whereas a low value may overemphasize weak similarity values in high-average connectivity networks. Thus, we recommend choosing an adaptive value determined based on the distribution of the similarity matrix. For this reason, the use of the average value is proposed. In order to evidence our choice, we illustrate, based on simulated data with different noise levels, the number of detected MSs as well the temporal accuracy as a function of *a* (Figures 5, 6, and 7 in the Supporting Information). In all cases, results show that the average value of the similarity matrix provides the exact number of MSs and a good temporal accuracy. Concerning the second parameter, *j*_*min*_, itwill depend on the way in which the connectivity matrices are computed. In the case where the connectivity is calculated over a time window that guarantees a sufficient number of data points, there is no need to set a minimal length for a state (*j*_*min*_ = 1). In contrast, when a connectivity matrix is computed at each millisecond, one may restrict the length of a state to be less than a specific number of milliseconds. As an example, Mheich, Hassan, Khalil, Berrou, & Wendling (2015) suggested to reject a brain state that has a time interval less than 30 ms. In our study, *j*_*min*_ was set to 1 time window in simulated data, Dataset 1 and Dataset 3, since an optimal sliding window was used. In Dataset 2, where a connectivity matrix based on the PLV metric was computed each millisecond, *j*_*min*_ was set equal to 30 ms, as proposed by Mheich et al. (2015).

Fourth, we are aware that spurious correlations caused by the problem of “source leakage” should be carefully considered. Here, we have adopted in each dataset the same pipeline (from data processing to network construction) used by the previous studies dealing with the same dataset. Thus, for the MEG dataset, the correction for source leakage was performed by the symmetric orthogonalization method proposed by Colclough et al. (2015). Using the same pipeline also helps to avoid influencing factors caused by changing the source connectivity method, the number of ROIs, the connectivity measure, or the sliding window length. By relying on previous studies (Hassan, Benquet, et al., 2015; Hassan et al., 2017; O’Neill, Tewarie, Colclough, et al., 2017), we provide appropriate input—already tested and validated—to the algorithm, regardless of how it was obtained. The source leakage issue was extensively discussed in a very recent review about M/EEG source-space networks (Hassan & Wendling, 2018).

In addition, a quantitative comparison presented in the paper was performed between our algorithm, K-means clustering as proposed by Allen et al. (2014), independent component analysis as proposed by O’Neill, Tewarie, Vidaurre, et al. (2017), and the consensus clustering algorithm as proposed by Rasero et al. (2017). Nevertheless, other strategies that showed accurate results in previous studies could be also investigated and compared, such as the use of a hidden Markov model (HMM), which models the cortical time series using a probabilistic generative model (Vidaurre et al., 2017), or the use of a multivariate autoregressive model (De Vico Fallani et al., 2008).

## ACKNOWLEDGMENTS

This work has received a French government support granted to the CominLabs excellence laboratory and managed by the National Research Agency in the “Investing for the Future” program under reference ANR-10-LABX-07-01. It was also financed by the Rennes University Hospital (COREC Project named conneXion, 2012-14). This work was financed by the Azm Center for Research in Biotechnology and Its Applications. GCO is funded by a Medical Research Council New Investigator Research Grant (MR/M006301/1). This study was also supported by the Future Emerging Technologies (H2020-FETOPEN-2014-2015-RIA under agreement No. 686764) as part of the European Union’s Horizon 2020 research and training program 2014–18. The study was also funded by the National Council for Scientific Research (CNRS) in Lebanon.

## AUTHOR CONTRIBUTIONS

Aya Kabbara: Conceptualization; Formal analysis; Methodology; Software; Validation; Visualization; Writing - Original Draft. Mohamad Khalil: Funding acquisition. Georges O’Neill: Data curation. Kathy Dujardin: Data curation. Youssof El Traboulsi: Methodology. Fabrice Wendling: Funding acquisition. Mahmoud Hassan: Conceptualization; Formal analysis; Supervision; Writing - Original Draft.

## FUNDING INFORMATION

Fabrice Wendling, Rennes University Hospital (http://dx.doi.org/10.13039/501100001665), Award ID: ANR-10-LABX-07-01. Mohamad Khalil, National Council for Scientific Research. Georges O’Neill, Medical Research Council, Award ID: MR/M006301/1. Fabrice Wendling, Hôpital de rennes1, Award ID: COREC Project named conneXion, 2012–14.

## TECHNICAL TERMS

Dynamic functional connectivity: Measurement of transient changes in the functional brain network across time.
Module: A subnetwork composed of strongly connected nodes that are weakly connected to the rest of the network.
Modular structure: The partition of the network into nonoverlapping modules.
Brain state: A network pattern representing time points that share some similar topological properties.
Functional connectivity: Statistical dependency between spatially separated brain regional time series.
Phase locking value: A measure used to quantify the statistical coupling, in term of phase synchrony, between two time series.
Resting state: An experimental paradigm in which the subject is asked to relax and to do nothing.
Task-related paradigm: An experimental paradigm in which the subject is asked to focus on a specific function.
EEG/MEG source connectivity: A method used to estimate functional brain networks at the cortical level using EEG/MEG sensors.
