# Supplementary material for: Detecting modular brain states in rest and task
Source: Netw Neurosci. 2019 Jul 1;3(3):878–901. doi: 10.1162/netn_a_00090 (PMC6663471; doi:10.1162/netn_a_00090)
Supplement: Supplementary file 1 [file netn-03-878-s001.pdf]

Kabbara, A., Khalil, M., O’Neill, G., Dujardin, K., El Traboulsi, Y., Wendling, F. & Hassan, M. (2019). Supporting information for "Detecting modular brain states in rest and task." *Network Neuroscience, 3*(3), 878–901. https://doi.org/10.1162/netn_a_00090

Supplementary materials for

Detecting modular brain states in rest and task

Kabbara A.[1, 2, 3], Khalil M.[2, 3], O’Neill G.[4], Dujardin K.[5, 6], El Traboulsi Y.[7], Wendling F.[1] and Hassan M.[1]

[1] Univ Rennes, LTSI - U1099, F-35000 Rennes, France

[2] Azm Center for Research in Biotechnology and its Application, EDST, Lebanese University, Lebanon

[3]Laboratoire CRSI, Faculté de génie, Université Libanaise, Liban

[4] Sir Peter Mansfield Imaging Centre, School of Physics and Astronomy, University of Nottingham, University Park, Nottingham, UK

[5] INSERM, U1171, F-59000 Lille, France

[6] CHU Lille, Neurology and Movement Disorders Department, F-59000 Lille, France

[7] LaMA-Liban, Lebanese University, Tripoli, Lebanon

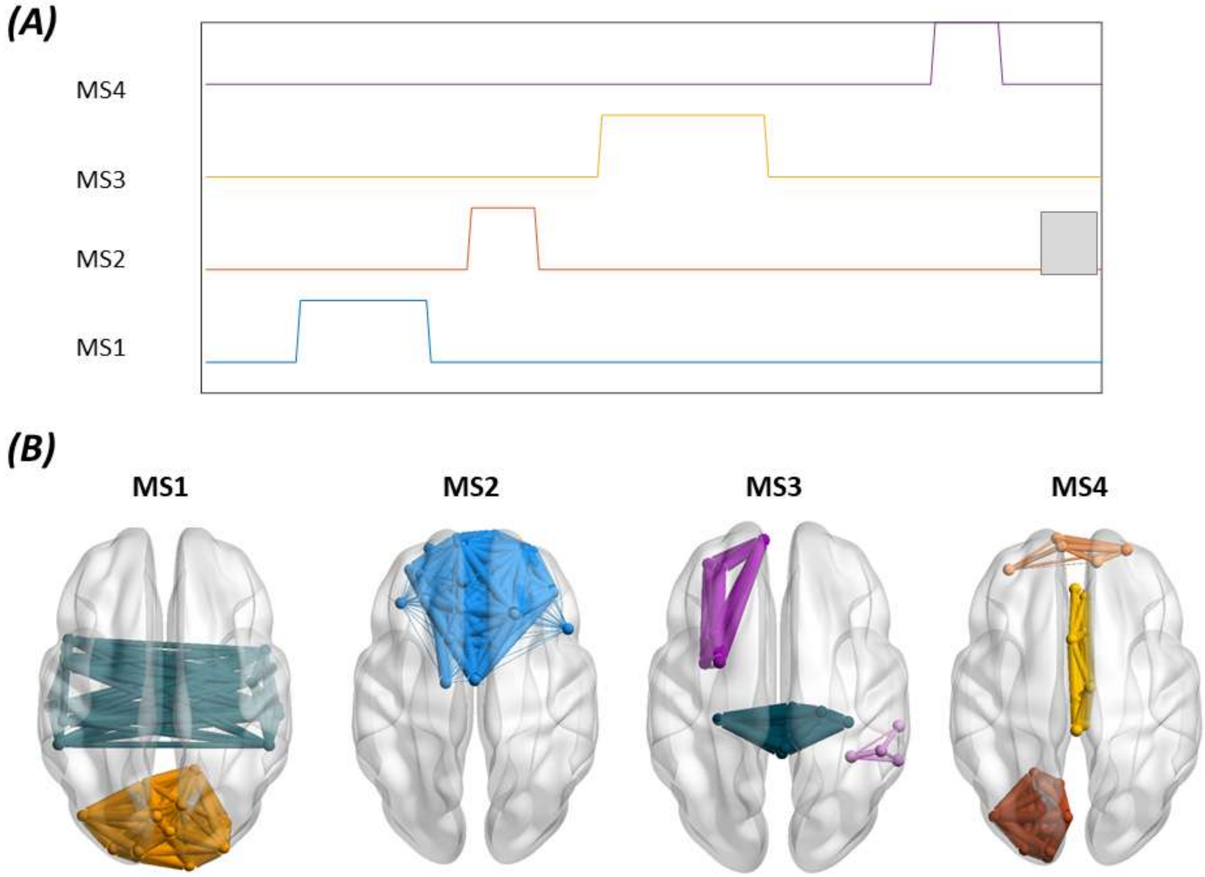

Figure 1. Results of the categorical method applied on simulated data ($STD_{noise} = 0.35$). A) the time course of the four modular structures reconstructed. The grey square indicates the missed time-window detection. B) 3D representation of the four modular structures states.

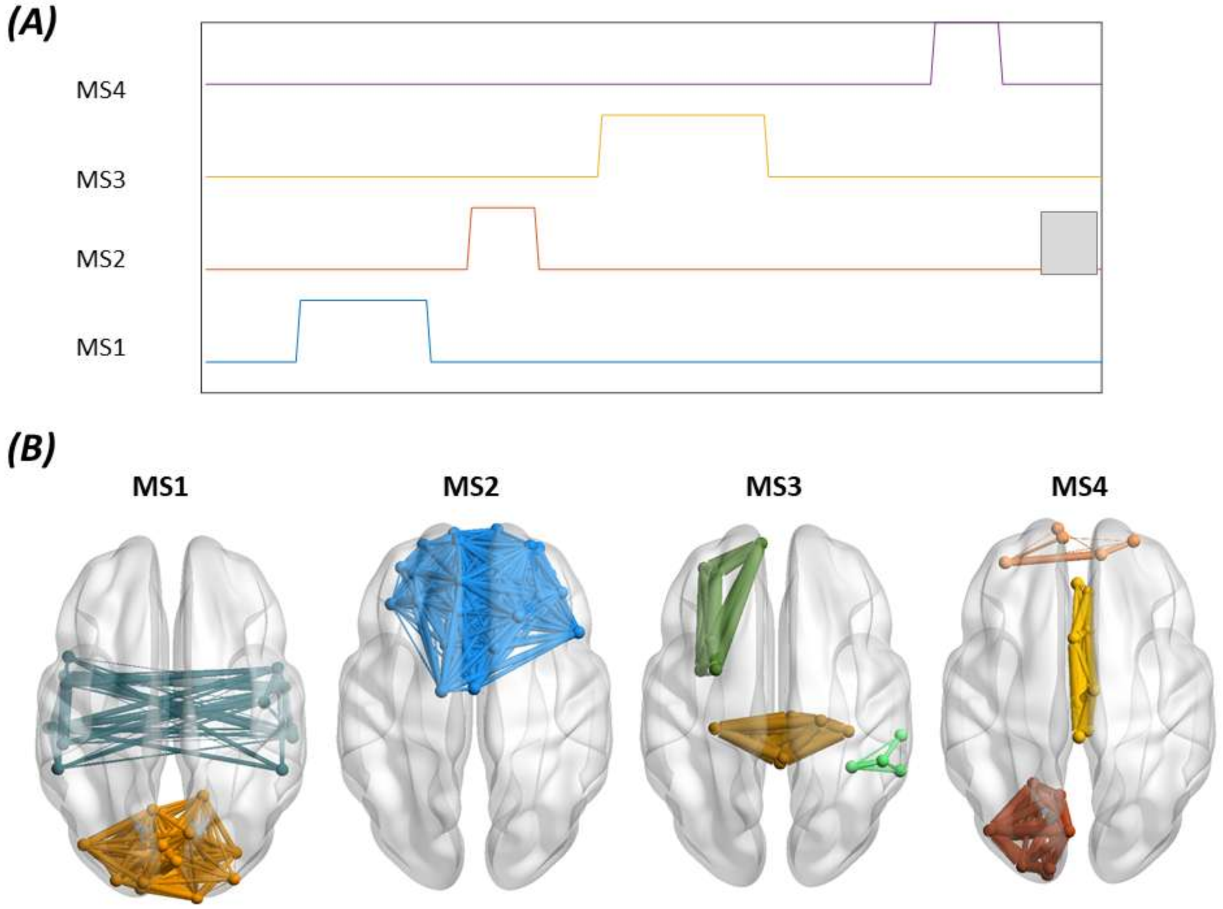

Figure 2. Results of the categorical method applied on simulated data ($STD_{noise} = 0.5$). A) the time course of the four modular structures reconstructed. The grey square indicates the missed time-window detection. B) 3D representation of the four modular structures states.

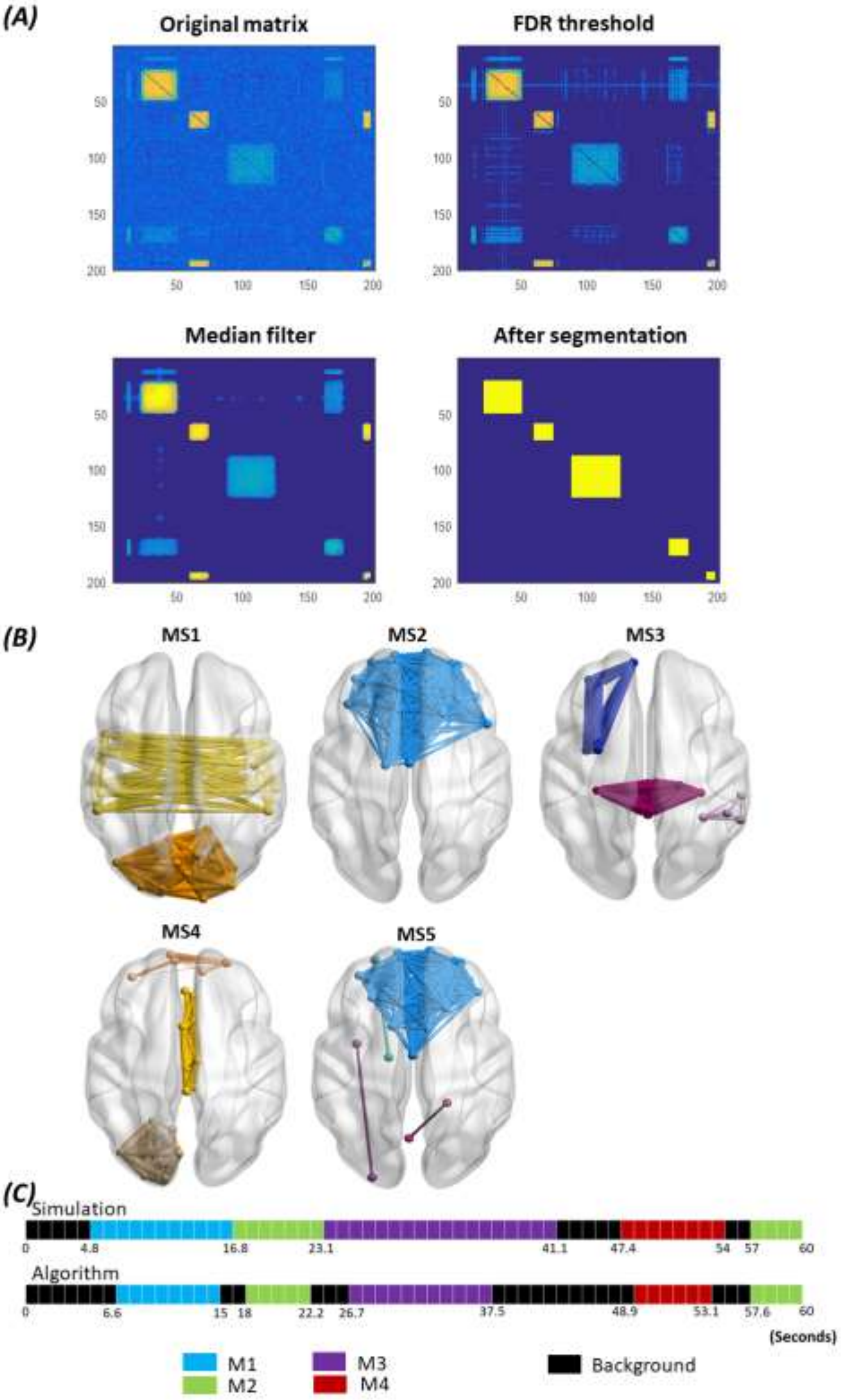

Figure 3. Results of the consecutive method applied on simulated data ($STD_{noise} = 0.35$). A) The different steps of the segmentation algorithm that ended to find 5 modular stuctures. B) The 3D representation of the six consecutive modular structures obtained. C) The difference between the simulated time axis and the obtained time axis.

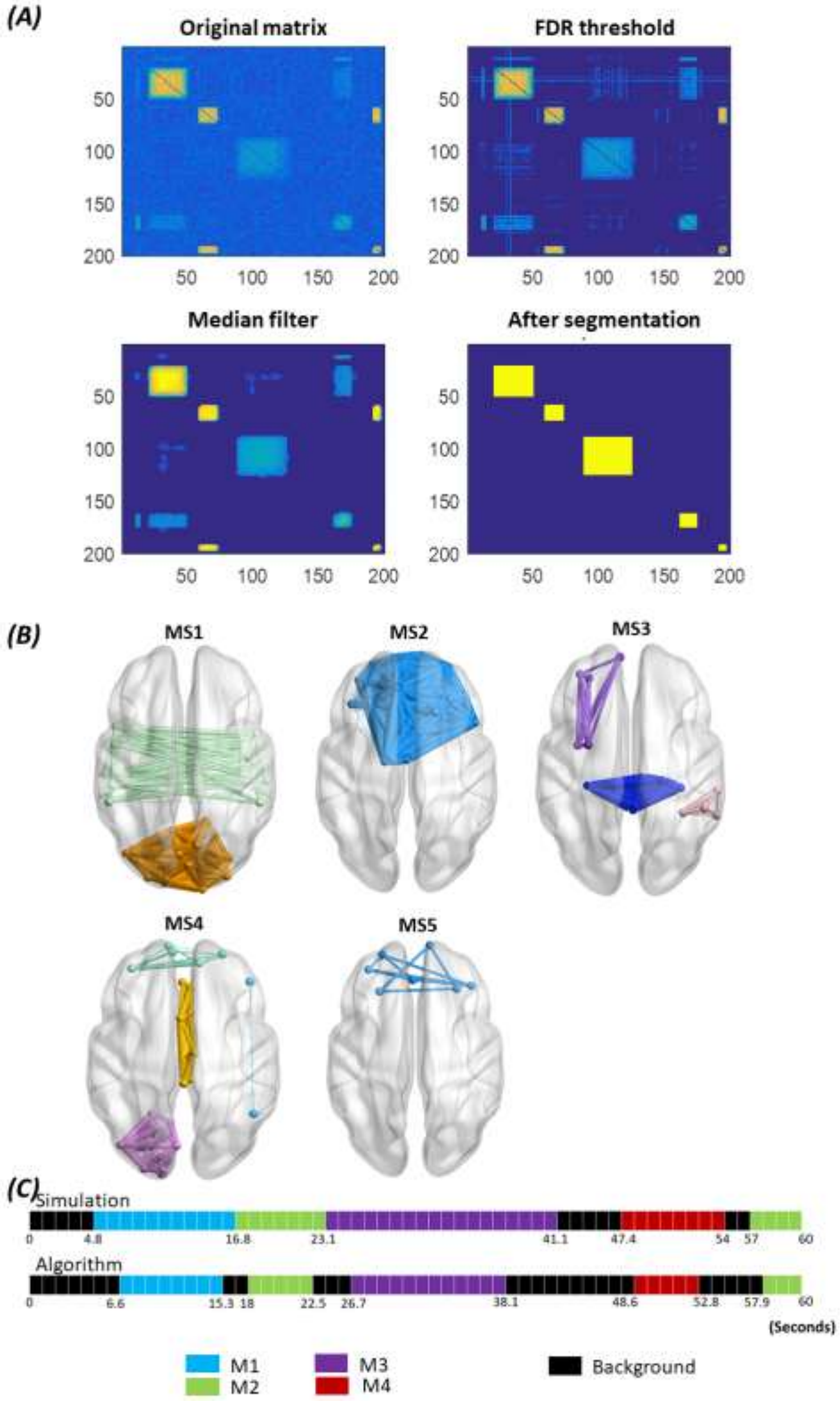

Figure 4. Results of the consecutive method applied on simulated data ($STD_{noise} = 0.5$). A) The different steps of the segmentation algorithm that ended to find 5 modular stuctures. B) The 3D representation of the six consecutive modular structures obtained. . C) The difference between the simulated time axis and the obtained time axis.

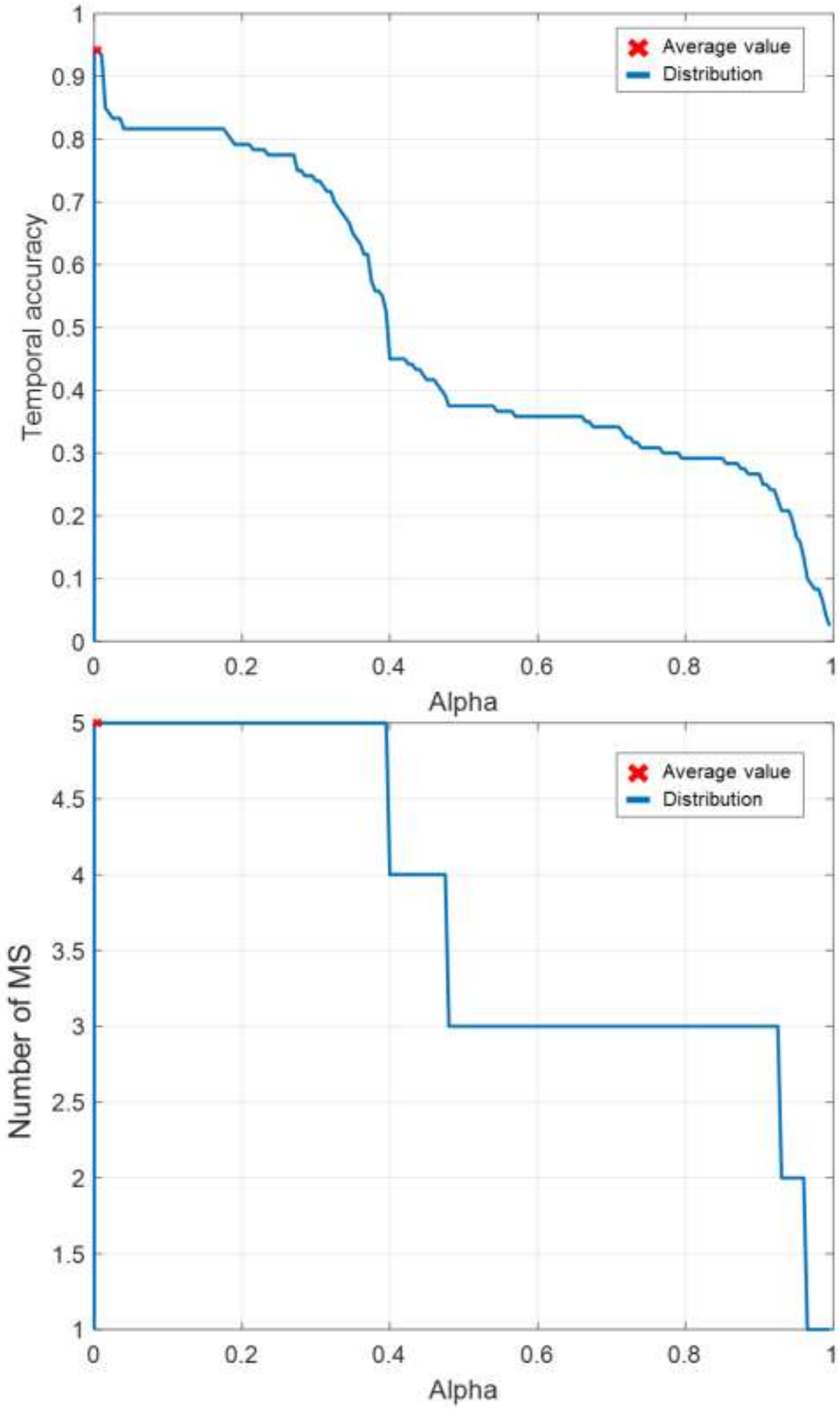

Figure 5. The temporal accuracy and the number of detected MS as functions of the accuracy parameter alpha ($STD_{noise} = 0.2$). The red marker corresponds to the average value.

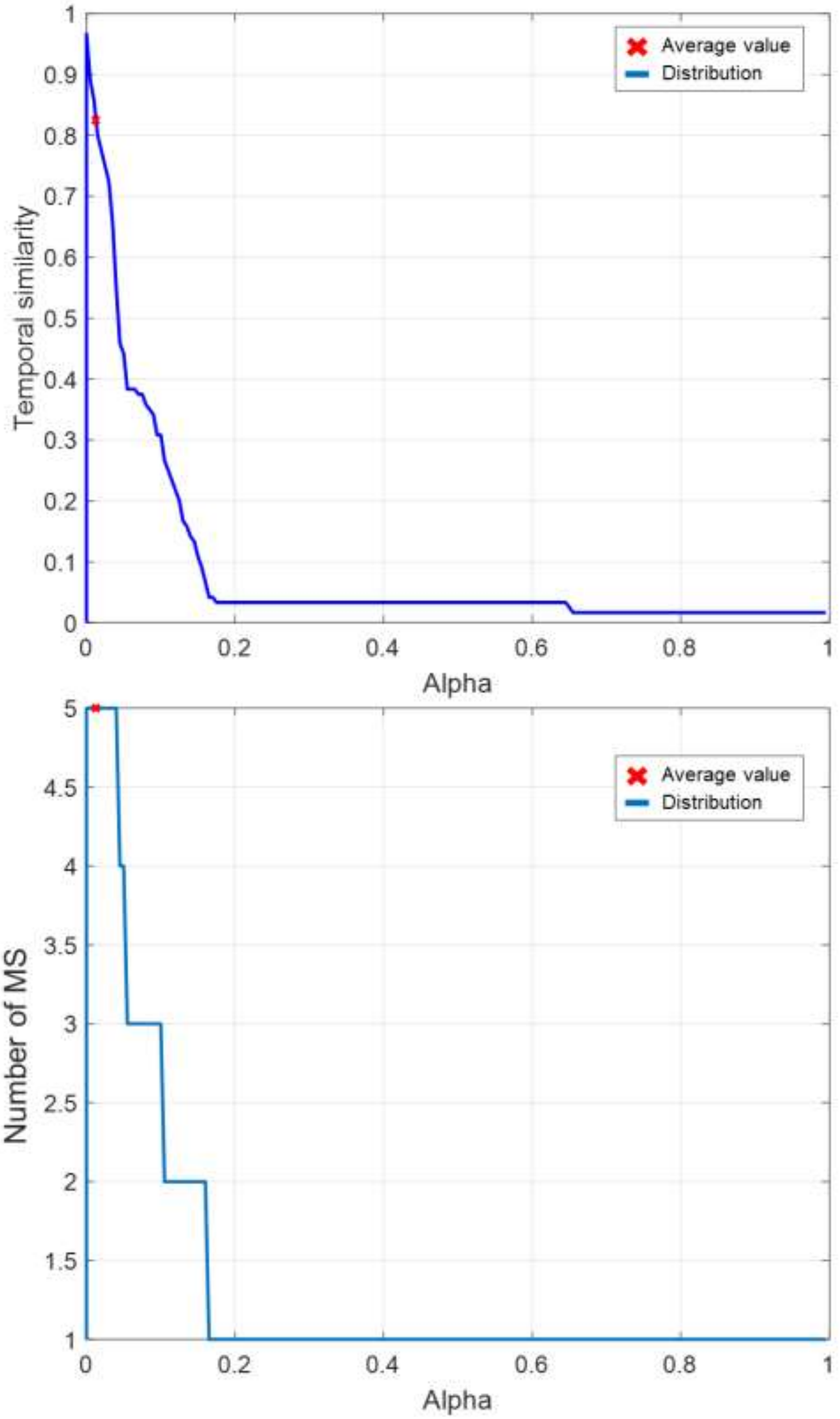

Figure 6. The temporal accuracy and the number of detected MS as functions of the accuracy parameter alpha ($STD_{noise} = 0.35$). The red marker corresponds to the average value.

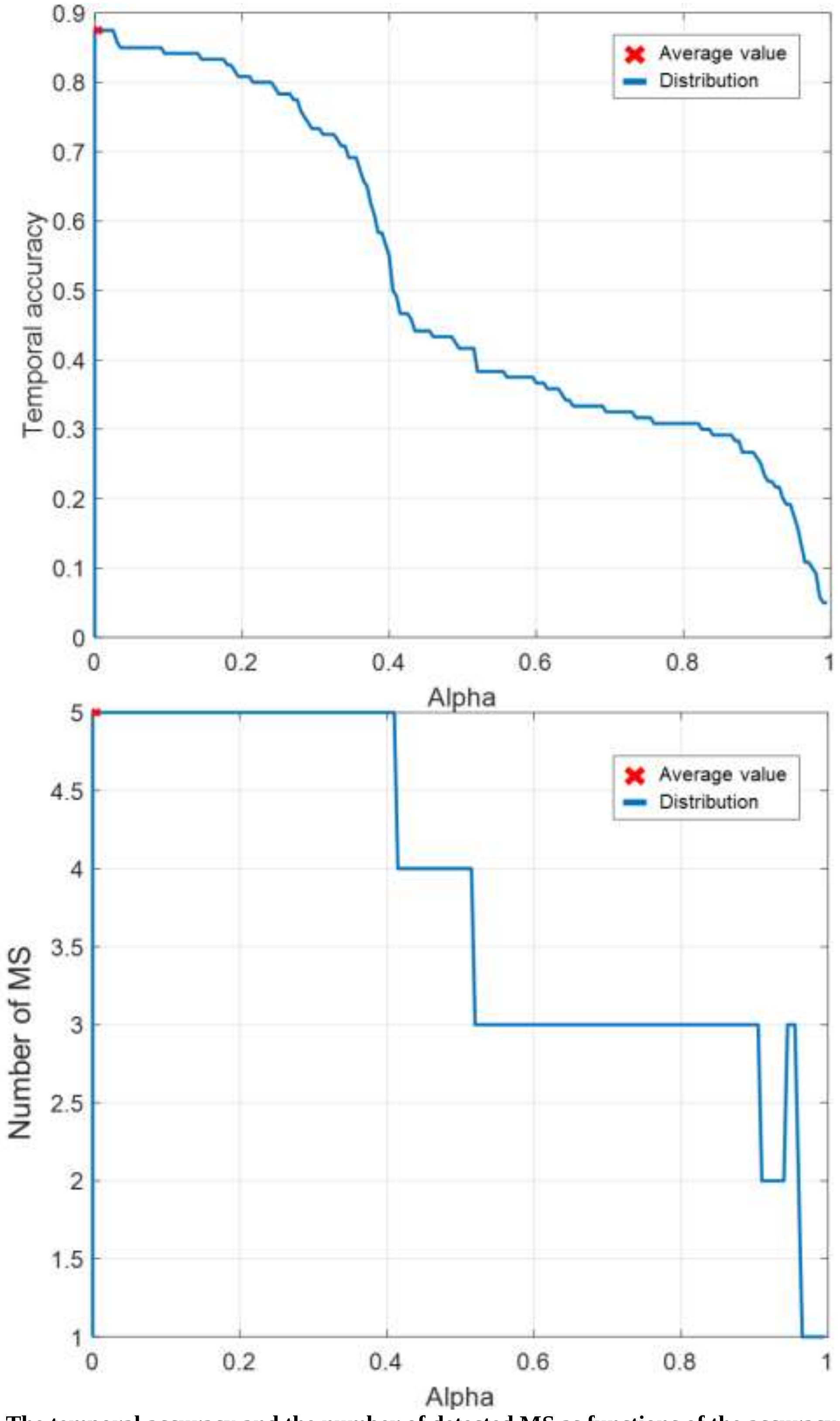

Figure 7. The temporal accuracy and the number of detected MS as functions of the accuracy parameter alpha ($STD_{noise} = 0.5$). The red marker corresponds to the average value.
